# Growth Study of Hierarchical Pore SSZ-13 Molecular Sieves with Improved CO_2_ Adsorption Performance

**DOI:** 10.3390/nano11123171

**Published:** 2021-11-23

**Authors:** Runlin Han, Yuxuan Tao, Liang Zhou

**Affiliations:** 1 School of Chemistry and Chemical Engineering, Jinggangshan University, Ji’an 343009, China; 2 School of Chemical Engineering, Dalian University of Technology, Dalian 116024, China; tyxlogo@163.com; 3 Jiangsu Province Key Laboratory of Fine Petrochemical Engineering, Changzhou University, Changzhou 213164, China

**Keywords:** SSZ-13, hydrothermal synthesis, hierarchical pore, molecular sieves, CO_2_ adsorption

## Abstract

SSZ-13, with a unique pore structure and excellent thermal stability, showed a potential application in the adsorption and catalysis industry. In this work, Al(NO_3_)_3_ was used as an Al source to study the performance and morphology of the zeolite. The zeolite was prepared with an unconventional process by adding an Al source before the structure-directing agent and base. When inorganic oxygen-containing anions were introduced into the unconventional synthesis system, the crystals of the zeolite conform to the unconventional growth mode. The zeolites with large crystals were assembled from small unit nanocrystals. Extending the reaction time, aging time and adding fluoride ions introduced a multistage pore structure on the surface of the molecular sieve, which improved the CO_2_ adsorption performance. When aging for 24 h, reaction for 96 h, and the amount of fluorine added was 0.05 (F/Si), the sample had the best hierarchical pore structure. The SSZ-13 molecular sieve with an added amount of 0.1 (F/Si) has the highest CO_2_ adsorption performance. The adsorption amount was 4.55 mmol/g at 1 bar, which is 20.4% higher than that of zeolite SSZ-13 prepared by the conventional process.

## 1. Introduction

China’s carbon dioxide emission is expected to peak around 2030, and it needs the government to make a great effort in emissions control and green technology innovation. CO_2_ capture is one of the most pressing issues in the world, which needs efficient and selective capture methods to decrease the emission of CO_2_ [1]. Zeolitic materials have been proved one of the most efficient materials of CO_2_ capture because of appropriate pore aperture dimensions, high thermal stability and ease of regeneration [2,3]. Researchers are searching for new types of zeolites and ways to decrease the cost of the synthesis process. SSZ-13 zeolite with three-dimensional eight-membered ring windows (0.38 nm × 0.38 nm) showed preferentially adsorption of CO_2_, which is meaningful to the CO_2_ capture industry. It has a chabazite (CHA) structure with a unique pore structure and excellent thermal stability [4].

SSZ-13, with excellent low-/high-temperature hydrothermal stability and high production selectivity, showed high potential in the catalytic industry due to the unique pore system [5]. In order to improve the performance of SSZ-13, preparation methods and recipes were studied persistently. It was found that the high silica SSZ-13 catalysts prepared with steam-assisted crystallization method exhibited a longer lifetime and comparative selectivity to ethylene and propene than the SSZ-13 obtained by conventional hydrothermal route during the methanol-to-olefins reaction [6]. Crystalline Cu/SSZ-13 particles with uniform and controllable sizes were synthesized by carefully controlling the concentration of zeolite seeds and the pH values to study the NH_3_-SCR reaction rate [7]. The Fe-SSZ-13 zeolite prepared with iron added during the preparation of the zeolite gel precursor showed good catalytic performance in the selective catalytic reduction of NOx with NH_3_ (NH_3_-SCR) [8]. SSZ-13 membrane has certain advantages over other zeolite membranes, including easy synthesis, good stability and insensitivity to moisture [9].

The pore structure of SSZ-13 is ideal for many industrial separation processes based on molecular sieving mechanisms, including natural gas purification [10]. Pham et al. studied the effect of ion type on the adsorption performance of SSZ-13. Protons and alkali-metal cations Li^+^, Na^+^, and K^+^, were investigated using adsorption isotherms of CO_2_. The Na-SSZ-13 have excellent CO_2_ capacity at ambient temperature and pressure, but the preparation of SSZ-13 needs 6 days [11]. The long synthesis time and expensive structure-directing agent (SDA) of the SSZ-13 molecular sieve make it difficult to prepare and high synthesis cost, which restricts its development [12]. Although many scientists have optimized the preparation method of SSZ-13 molecular sieve by seed induction, the use of alternative template agents, the addition of chemical additives or the use of additional devices, and so on, reduced the preparation cost or shortened the synthesis time [13]. The different order of adding materials will affect the degree of dissolution of each material in the synthetic solution and change the degree of homogenization of the synthetic gel. It was found that the addition order of synthesis mixture components influenced starting gel pH and mix rheology. It affected the crystallinity and phase purity of SAPO catalysts [14]. However, there is little research on the feeding sequence of the SSZ-13 molecule sieve.

In this work, in order to decrease the synthesis time and adjust the zeolite pore structure, an unconventional feeding sequence with Al source added first was used for the first time, as far as we know. Aluminum nitrate was used as the aluminum source to study the growth process and morphology of SSZ-13 zeolite in detail. The materials were characterized with SEM, TEM, XRD and N_2_ adsorption. The CO_2_ adsorption performance was compared with the SSZ-13 prepared by the conventional process.

## 2. Experimental

### 2.1. Synthesis of SSZ-13

The unconventional synthesis process was conducted as follows: Al(NO_3_)_3_ (Damao, Tianjin, China) was dissolved in deionized water with stirring at 25 °C. Then *N*,*N*,*N*-trimethyl-1-adamantanammonium hydroxide (TMAdaOH, 25 wt.%, Alladin, Shanghai, China) was added in the solution as SDA and stirred for 30 min. NaOH (Kemiou, Tianjin, China) was mixed in the solution. The colloidal silica (40 wt.%, Aldrich, Burlington, MA, USA) was added in slowly after NaOH was dissolved completely. Then the solution was transferred to a Teflon reaction kettle for reaction at 433 K for a certain time, and the synthesis conditions were listed in Table 1. Sample S-15 for comparison was synthesized with the conventional process. When the SDA and NaOH were mixed uniformly, the Al source was added to the solution. The difference in feeding sequence between conventional and unconventional processes is shown in Scheme S1.

### 2.2. Characterization

X-ray powder diffraction (XRD, XRD-7000S, Shimadzu, Japan) was utilized to analyze the crystal structure with Cu Kα radiation (40 kV, 40 mA, λ = 1.5418 Å). The morphology of the zeolite was analyzed via scanning electron microscopy (SEM, Nova Nano SEM 450, FEI, Hillsboro, OR, USA) after the samples were coated with gold sputtering. The samples were also tested with transmission electron microscopy (TEM, Tecnai F30, FEI, Hillsboro, OR, USA) at the acceleration voltage of 300 kV. The CO_2_ adsorption performance was analyzed with a specific surface area aperture analyzer (V-Sorb-2800P, Gold APP Instruments, Beijing, China) at 298 K, while N_2_ adsorption/desorption test was conducted at 77 K.

## 3. Results and Discussion

### 3.1. Effect of Reaction Time on the Sample Structure

**Figure 1:**
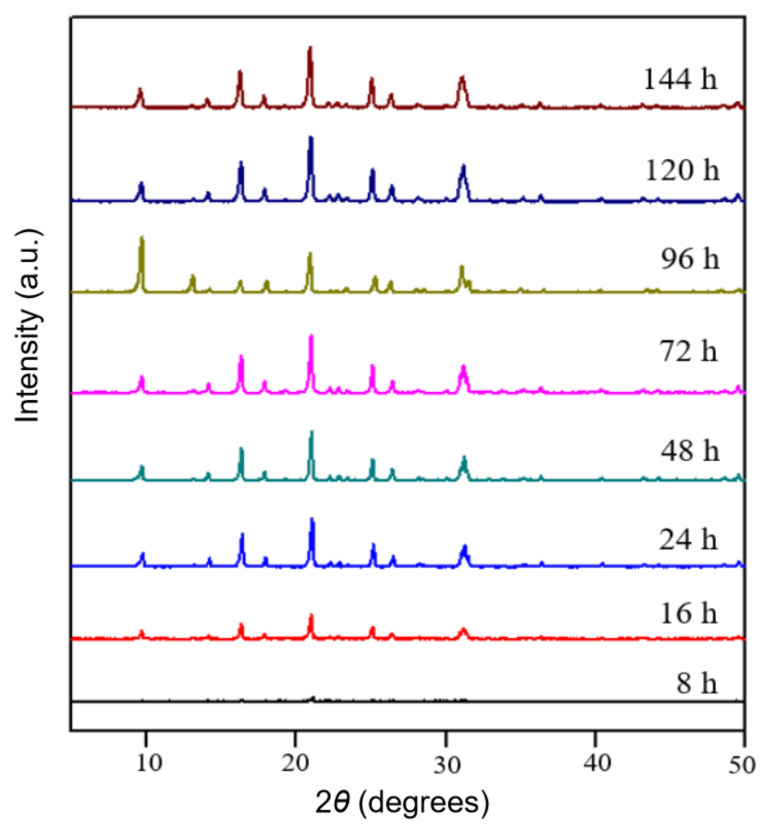
XRD patterns of samples prepared at various reaction times.

The powder X-ray diffraction test was carried out on the samples prepared at different reaction times, as shown in Figure 1. There were almost no characteristic peaks appeared at 2θ = 13.5°, 20.5°, 23.5° and 31.5° when the samples reacted for 8 h. As the reaction went on, the intensity of the characteristic peak of CHA of the samples gradually increased. The characteristic peak intensity reached the highest, except for the sample S-6, which reacted for 96 h, and the peak intensity of the product did not change with time.

**Figure 2:**
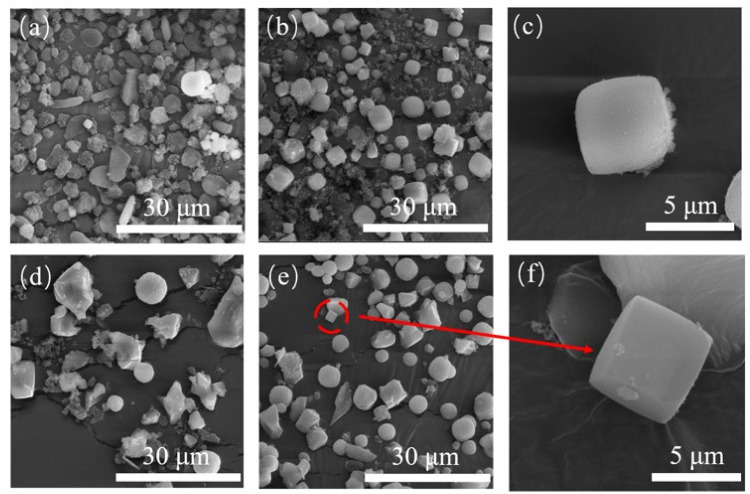
SEM diagrams of S-1(**a**), S-2 (**b**,**c**), S-3 (**d**) and S-4 (**e**,**f**).

Figure 2 shows the SEM images of the products after reaction for 8 h, 16 h, 24 h and 48 h. When the reaction time was 8 h, most of the particles were amorphous, and a small number of cubic crystals could be found, which corresponds to the tiny XRD characteristic peaks in the XRD patterns. As the reaction went on, the amorphous particles decreased gradually. When the reaction reached 72 h, as shown in Figure 2a, the products were mainly spherical or cubic large crystals assembled by small cubic crystals. The amorphous and fragmental crystals completely disappeared, and their sizes were distributed between 5 and 10 μm. Combined with XRD data and SEM images, the synthesis time of the SSZ-13 molecular sieve was shortened to 3 days with the unconventional process.

**Figure 3:**
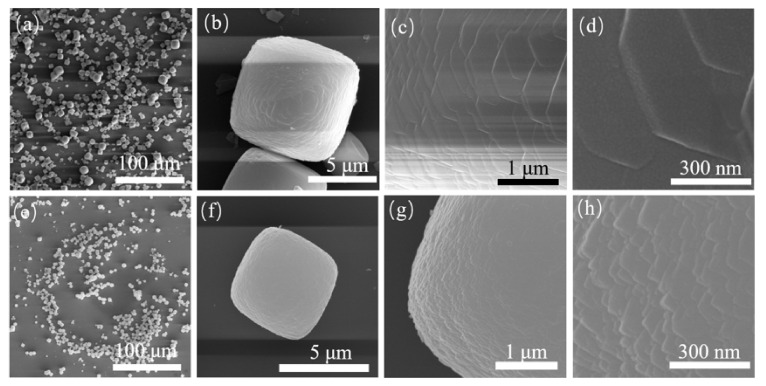
SEM diagrams of S-5 (**a**–**d**) and S-6 (**e**–**h**).

**Figure 4:**
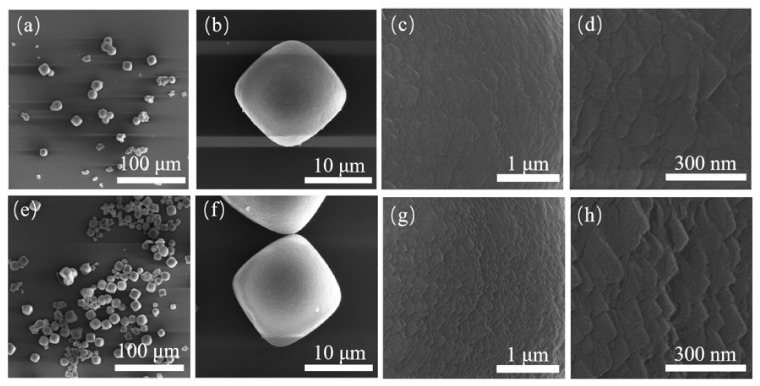
SEM diagrams of S-7 (**a**–**d**) and S-8 (**e**–**h**) with different magnifications.

Crystals assembled with small cubic crystals can be obtained using aluminum nitrate as the Al source. The reaction time of the samples was extended to 4, 5 and 6 days. Their SEM images are shown in Figure 3 and Figure 4. When the sample was reacted to 16 h, the surface of the product was a roughly aggregated sphere composed of small particles. Combined with the XRD patterns with the characteristic peak of CHA, they can be identified as CHA nuclei or extremely small CHA crystals. As the reaction went on, amorphous substances in the products decreased, and spherical particles with different sizes gradually increased. Traditional CHA cubic crystals with smooth surfaces can be observed (see Figure 3e, Figure 4a,e). After reaction for 72 h, the products were mainly cubic crystals, and the unit crystals involved have been completely transformed into small cubic crystals, and these unit crystals were assembled and superposed together in the way of crystal plane co-orientation. With the extension of reaction time, the characteristic peak strength of the sample decreased slightly at 4 days after reaction, but it had a larger peak strength at (101) plane (2θ = 7.7°). According to previous literature, it was indicated that the crystal morphology of the sample was closest to the cube type, which was also confirmed by Figure 3f. As the reaction time went on, the crystal size of the sample increased gradually.

**Figure 5:**
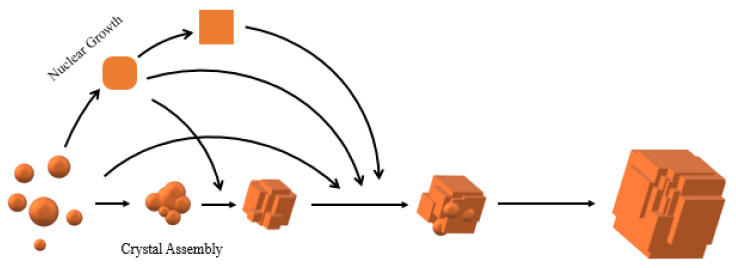
Growth process of SSZ-13 crystal with the unconventional process.

**Figure 6:**
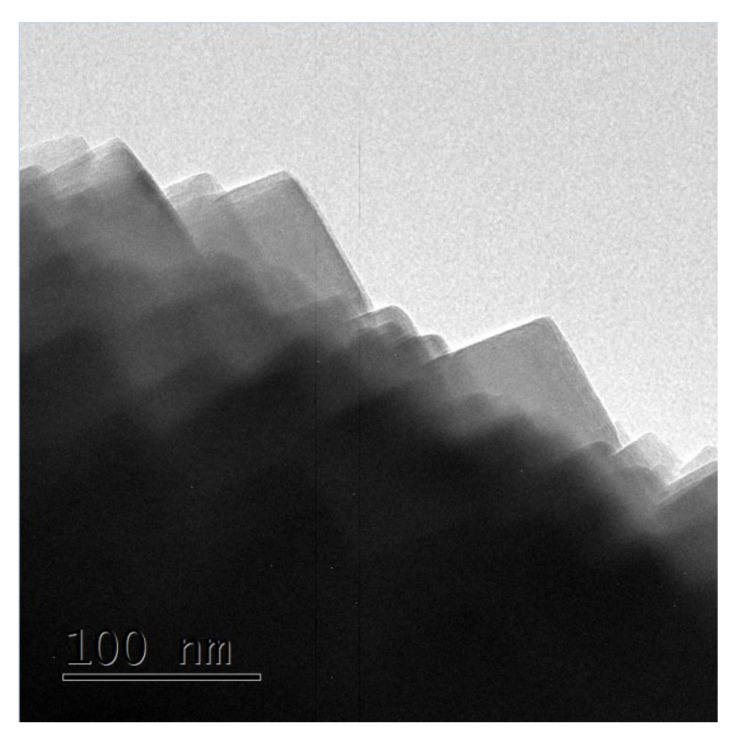
TEM image of sample S-6.

The above phenomena indicated that the zeolite grew from spherical crystal to rounded crystal and then to cubic crystal during the assembly of the unit crystal. Regardless of the shape of the unit crystal, it participated in the assembly process. The specific crystal growth diagram is shown in Figure 5. With the extension of reaction time, it can be seen from the TEM images of sample S-6 in Figure 6 that there was no obvious pore structure inside the crystal, and there were many staggered crystals on the sample surface. Some cracks appeared in the interlaced part of the sample surface.

**Figure 7:**
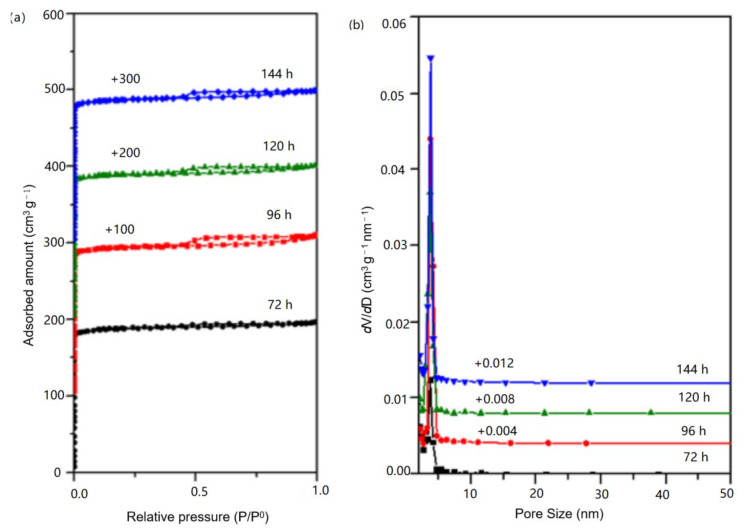
(**a**) N_2_ adsorption isotherm (**b**) pore size distribution of sample S-5, S-6, S-7 and S-8.

Then the samples S-5, S-6, S-7 and S-8 were tested for N_2_ adsorption/desorption. Figure 7 shows the N_2_ physical adsorption curve and Barrett–Joyner–Halenda (BJH) pore size distribution of the samples. Table 2 shows the N_2_ physical adsorption data of the samples. As can be seen from Figure 7a, within the range of 10^−6^ < P/P^0^ < 0.01, the adsorption curve rose rapidly and reached saturation after the adsorption amount reached 190 mL/g, which was caused by the adsorption of N_2_ by the micropores in the sample. S-6, S-7 and S-8 all had hysteresis loops at P/P^0^ = 0.4~1.0, among which S-6 had the largest hysteresis loop. Additionally, the hysteresis loops gradually shrunk with the reaction. The mesoporous pore size distribution diagram in Figure 7b showed that the crystal pore sizes of the samples were mainly distributed at 2–5 nm. However, the peak of the distribution curve of the S-5 sample was significantly lower than that of the other three samples, indicating that the mesoporous content of the S-5 sample was small. Table 2 shows that the sample S-6 had the largest specific surface area, total pore volume, micropore volume and mesoporous volume, which were 644 m^2^/g, 0.33 mL/g, 0.04 cm^3^/g and 0.29 cm^3^/g, respectively, and the mesoporous proportion reached 12.12%. All the above data indicated that SSZ-13 with high N_2_ adsorption performance could be obtained when Al(NO_3_)_3_ was used as the Al source and the optimal reaction time was 4 days.

### 3.2. Effect of Aging Time on the SSZ-13 Molecular Sieve Structure

Figure S1 showed the XRD patterns of the samples S-6, S-9, S-10 and S-11. It can be seen from the images that, with the extension of aging time, the positions of the diffraction peaks of the samples did not change. They were all in line with the characteristic peaks of CHA type, indicating that the crystal type and crystallinity of the samples did not change with aging time. Figures S2 and S3 were SEM images of the samples with different aging times. When the aging time was extended to 12 h, the assembly phenomenon of small crystals was more obvious, and the crystal size was increased. The increase of aging time led to the existence of more nucleic precursors in the synthetic gel, and the number of unit crystals increased at the initial reaction stage. Spherical crystals with rougher surfaces and larger sizes were formed because more unit crystals participated in the crystal assembly process. When the aging time was 18 h, the crystal size further increased to 10 μm. Moreover, two large crystals grew mutually, which indicated that the crystal assembly phenomenon not only occurred between the unit crystals but also between the spherical crystals. After magnification of 400,000 times, it was observed that the unit crystals of samples S-9 and S-10 dissolved each other. It was speculated that, due to the increase of the crystal size, the crystal could no longer grow by the non-classical growth mechanism. It became classical interactive growth with adjacent unit crystals. When the aging time was up to 24 h, the crystal size decreased sharply to only 2–4 μm with uniform size, and the dissolution of unit crystal was not obvious. The samples S-6, S-9, S-10 and S-11 were tested for N_2_ physical adsorption, and the results are shown in Figure S4. The rapidly increased adsorption capacity of all samples in the range of 10^−6^ < P/P^0^ < 0.01 meant the typical N_2_ adsorption curve of microporous materials. S-6 and S-11 had obvious hysteresis loops at P/P^0^ = 0.4~1.0, indicating certain mesoporous structures. The mesoporous pore size distribution of S-6 and S-11 showed that the number of pores in the range of 2–5 nm was significantly higher than that of S-9 and S-10. Combined with the SEM images of Figures S2f and S3c, the dissolution and mutual growth of the surface unit crystals of the sample destroyed the mesoporous structure on the surface of the crystal. Table S1 provides their N_2_ physical adsorption data, and their specific surface areas reached more than 620 cm^2^/g, which indicated that assembled SSZ-13 crystals, formed through non-classical growth mode, had a great advantage in specific surface areas. However, both samples S-6 and S-11 had relatively high micropore volume and large mesoporous desorption area, accounting for 12.12% and 12.5% of the total pore volumes, respectively. It indicated more mesoporous structures, which is consistent with the results of the pore size distribution curve. The smaller the size of the molecular sieve, the greater its potential application value in the fields of adsorption and catalysis. Combined with the SEM images, sample S-11 had a certain mesoporous structure and small particle size. The synthesis condition was studied further.

### 3.3. Effect of Sodium Fluoride Addition on the Performance of SSZ-13 Molecular Sieve

**Figure 8:**
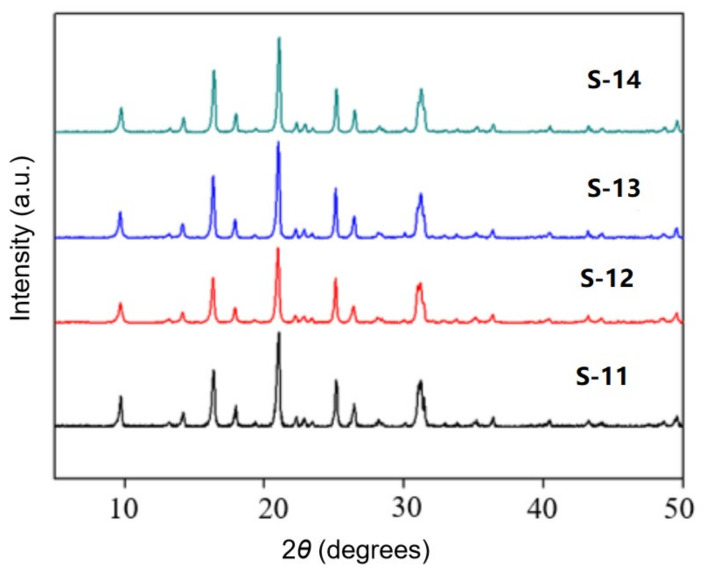
XRD patterns of samples S-11, S-12, S-13 and S-14.

**Figure 9:**
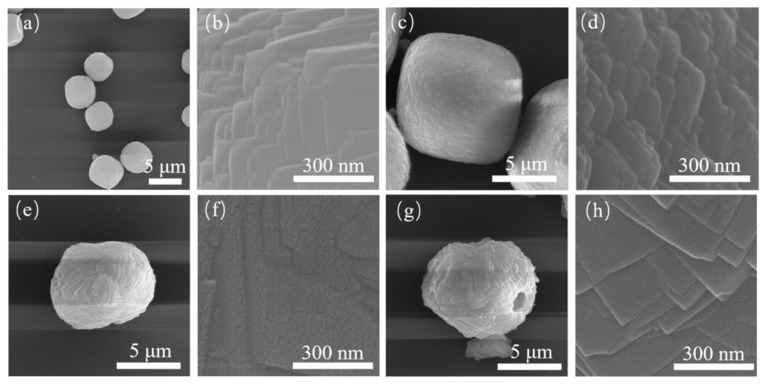
SEM diagrams of S-11 (**a**,**b**), S-12 (**c**,**d**), S-13 (**e**,**f**) and S-14 (**g**,**h**).

**Figure 10:**
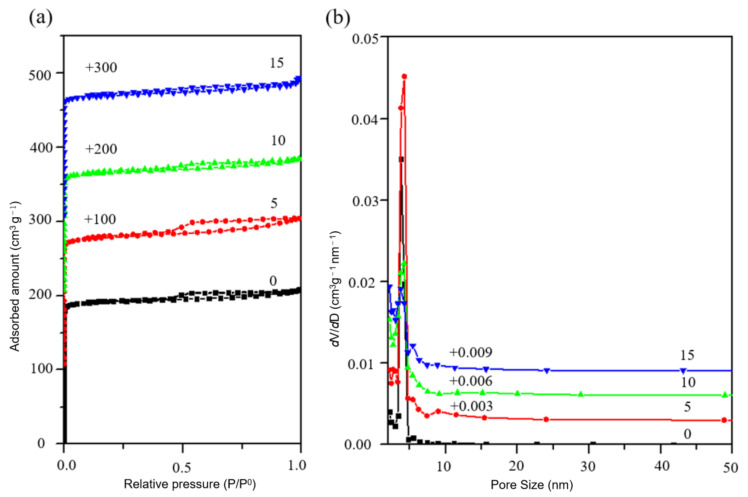
(**a**) N_2_ adsorption isotherm and (**b**) pore size distribution of sample S-11, S-12, S-13 and S-14.

The samples S-11, S-12, S-13 and S-14 were characterized by X-ray diffractometer, as shown in Figure 8. The position and intensity of the diffraction peak of the crystal did not change significantly, indicating that the addition of fluoride did not affect the CHA crystal form of the products. By observing the SEM figures of the samples (Figure 9), it was found that the size of the samples increased with the increase of fluoride addition, and the crystal size of the samples of S-12 and S-13 reached 5 μm. When the additive amount reached 10, the crystal intergrowth occurred. When the addition amount is 15, even the phenomenon of multiple crystal staggered superposition appeared due to the strong activation between mineralizer fluoride and silicon aluminate, which improved the degree of saturation of zeolite. Then the samples were subjected to N_2_ physical adsorption test, and their adsorption/desorption isotherm and pore size distribution curves were shown in Figure 10. According to the adsorption and desorption isotherms, the samples S-11 and S-12 have obvious hysteresis loops at P/P^0^ = 0.4~1.0. It indicated that both samples have certain mesoporous structures, and the hysteresis loops of S-12 are more obvious. By comparing the pore size distribution diagram, it can be seen that all the four samples were distributed at the pore size of 2–5 nm, but only the samples S-11 and S-12 were more distributed. The distribution of the samples at 4 nm slightly increased with the addition of fluoride, but the distribution rapidly decreased with the continuous increase of fluoride. The addition of described fluorine introduced mesopores of about 4 nm into the SSZ-13 molecular sieve.

In the experiment of the previous section, SSZ-13 with a certain mesoporous structure was prepared by using aluminum nitrate and prolonging the aging time. According to the study of Zhu et al. [15], SSZ-13 with a double microporous structure can be prepared by adding an F ion into the synthetic system. An aqueous solution of 40 wt.% NH_4_F was also used to prepare etched NH_4_-SSZ-13 and obtain hierarchical derivatives [16]. Therefore, F was added to the reaction system in the form of sodium fluoride, and the influence of the amount of NaF on the product was investigated in this work.

**Figure 11:**
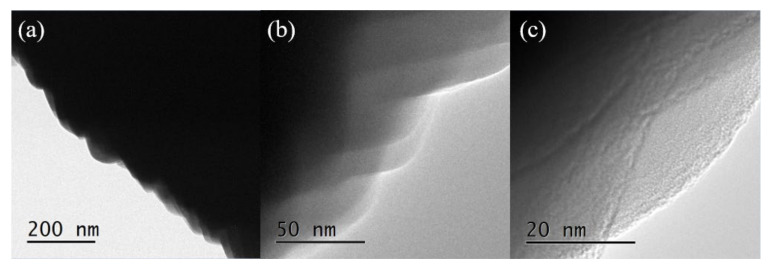
TEM images of sample S-12. (**a**–**c**) are the TEM images of sample S-12 with different magnifications and sites.

Table 3 shows the adsorption data of each sample. It can be seen that, with the introduction of fluorine into the synthesis system, the laminated assembly phenomenon of the samples is more obvious while the specific surface area decreases. This may be due to the gradual increase in crystal size. Among them, sample S-12 has the largest mesoporous volume and desorption cumulative pore internal surface area, which are 0.06 cm^3^/g and 56.74 m^2^/g, respectively. Combined with the TEM picture of sample S-12 (Figure 11b), it can be seen that there is no obvious mesoporous structure inside the crystal (Figure 11a). However, there are indeed some nanoscale pores near the surface of the sample crystal (as shown in Figure 11c), which is consistent with the results of the N_2_ adsorption isotherm and pore size distribution of the sample. The above results indicated that the optimal ratio of F/Si was 0.05.

### 3.4. CO_2_ Adsorption Performance of SSZ-13 Molecular Sieve

**Figure 12:**
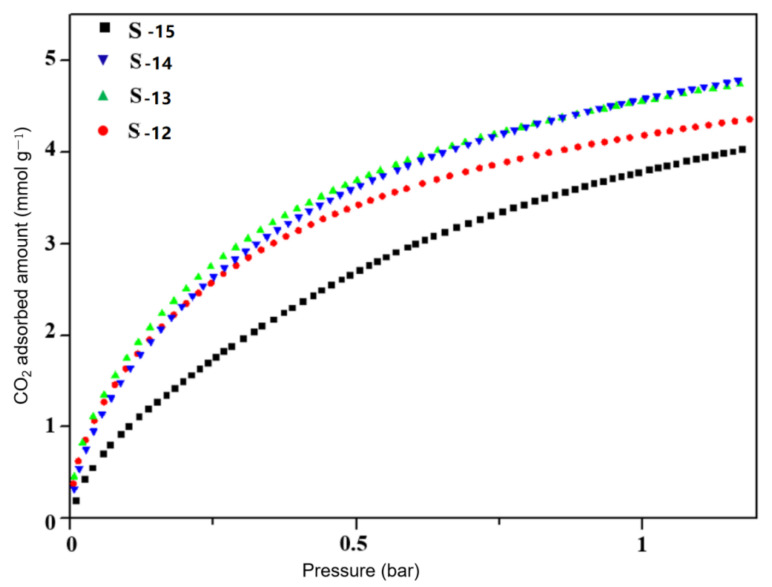
CO_2_ adsorption isotherm of samples S-12, S-13, S-14 and S-15.

In order to compare the performance of samples prepared with the unconventional process, sample S-15 was prepared with the conventional process, as described in the experimental section. The CO_2_ adsorption performance of samples S-12, S-13, S-14 and S-15 was tested at 273 K and are shown in Figure 12. It can be seen from the figure that the CO_2_ adsorption capacity of SSZ-13 molecular sieves prepared with aluminum nitrate and sodium fluoride was significantly higher than that of the SSZ-13 molecular sieve prepared by the conventional process at various pressures. The adsorption capacities of samples S-12, S-13 and S-14 were close to each other at low adsorption pressure (0–0.3 bar). Among them, S-13 had the largest adsorption capacity of CO_2_, which was 2.74 mmol/g at the pressure of 0.25 bar, much higher than the 1.77 mmol/g of SSZ-13 synthesized by the conventional process. With the increase in adsorption pressure, the adsorption capacity of all samples increased. The adsorption capacity of samples S-13 and S-14 was still significantly higher than that of sample S-15. When the pressure reached 1 bar, the adsorption capacities of S-12, S-13, S-14 and S-15 were 4.19, 4.55, 4.59 and 3.78 mmol/g, respectively. The above results indicated that the SSZ-13 molecular sieve prepared with the unconventional process had significantly better adsorption performance than the SSZ-13 molecular sieve prepared by the conventional process at various pressures, especially under low-pressure conditions. The adsorption capacity of S-13 at 1 bar was maintained at a high level, which was increased by 20.4% compared with the adsorption capacity of S-15. It was also higher than the CO_2_ adsorption capacity of H-SSZ-13 (3.75 mmol/g) and Cu-SSZ-13 (3.98 mmol/g) at the same pressure and temperature [17]. Therefore, the unconventional process endowed the SSZ-13 uniform structure and high adsorption performance because of the fine assembly of unit crystals with superior CO_2_ adsorption sites.

## 4. Conclusions

In this work, the samples prepared with the unconventional process were characterized by XRD, SEM, BET and TEM. The crystal formation process and crystal morphology of SSZ-13 were investigated with aluminum nitrate used as the Al source. On this basis, the effects of aging time and the amount of fluoride on the product were further optimized. SSZ-13 was found to be crystallized by a non-traditional growth mechanism and assembled from a spherical unit crystal. Additionally, the spherical crystal grew into a cubic crystal as the reaction proceeds, both processes occurring simultaneously. The final SSZ-13 also had a certain mesoporous structure. After 4 days of reaction, the product had the most mesoporous structure, accounting for 12.12% of the total pore volume, and the specific surface area reached 644 m^2^/g. After adding fluoride ions to the synthesis system, it was found that fluoride ions can improve the superposition and interlacing phenomenon of the sample crystal. When the ratio of F/Si was 0.05, mesoporous structures of the SSZ-13 molecular sieve accounted for 18.75% of the total pore volume, and the pore area was 56.74 m^2^/g according to the N_2_ physical adsorption test. The best adsorption performance was obtained when the fluoride content was 0.1, and the adsorption capacity was 2.74 mmol/g at 0.25 bar, which was 54.8% higher than that of SSZ-13 prepared with the unconventional process. When the pressure was 1 bar, the CO_2_ adsorption capacity of S-13 was 4.55 mmol/g, which was also 20.4% higher than that of SSZ-13 prepared with the conventional process. Therefore, the SSZ-13 prepared with the unconventional process showed high CO_2_ adsorption capacity, which had potential application in CO_2_ separation and capture.

## Figures and Tables

**Table 1:** Synthesis conditions of SSZ-13 molecular sieves.

Sample	Solution Composition SDA:NaOH:Al:Si:H_2_O:F	Al Source	T (°C)	t (h)	Aging Time (h)
S-1	20:20:5:100:4400:0	Al (NO_3_)_3_	160	8	6
S-2	20:20:5:100:4400:0	Al (NO_3_)_3_	160	16	6
S-3	20:20:5:100:4400:0	Al (NO_3_)_3_	160	24	6
S-4	20:20:5:100:4400:0	Al (NO_3_)_3_	160	48	6
S-5	20:20:5:100:4400:0	Al (NO_3_)_3_	160	72	6
S-6	20:20:5:100:4400:0	Al (NO_3_)_3_	160	96	6
S-7	20:20:5:100:4400:0	Al (NO_3_)_3_	160	120	6
S-8	20:20:5:100:4400:0	Al (NO_3_)_3_	160	144	6
S-9	20:20:5:100:4400:0	Al (NO_3_)_3_	160	96	12
S-10	20:20:5:100:4400:0	Al (NO_3_)_3_	160	96	18
S-11	20:20:5:100:4400:0	Al (NO_3_)_3_	160	96	24
S-12	20:20:5:100:4400:5	Al (NO_3_)_3_	160	96	24
S-13	20:20:5:100:4400:10	Al (NO_3_)_3_	160	96	24
S-14	20:20:5:100:4400:15	Al (NO_3_)_3_	160	96	24
S-15	20:20:5:100:4400:0	Al (NO_3_)_3_	160	48	6

**Table 2:** N_2_ physical adsorption data of S-5, S-6, S-7 and S-8.

Sample	S_BET_ (m^2^/g)	Total Pore Volume ^1,3^ (mL/g)	Mesoporous Volume ^2^ (cm^3^/g)	Micropore Volume ^1^ (cm^3^/g)	External Surface Area ^1^ (m^2^/g)	
S-5	626	0.30	0.02	0.28	29.12	
S-6	644	0.33	0.04	0.29	27.35	
S-7	627	0.31	0.03	0.28	24.49	
S-8	621	0.31	0.03	0.28	30.13	

^1^ Calculated with t-plot method. ^2^ Calculated with BJH method. ^3^ P/P^0^ = 0.9918, 0.9885, 0.9906, 0.9947.

**Table 3:** N_2_ physical adsorption data of S-11, S-12, S-13 and S-14.

Sample	S_BET_ (m^2^/g)	Total Pore Volume ^1,3^ (mL/g)	Mesoporous Volume ^2^ (cm^3^/g)	Micropore Volume ^1^ (cm^3^/g)	Desorption Cumulative Surface Area (m^2^/g)
S-11	642	0.32	0.04	0.28	31.77
S-12	601	0.32	0.06	0.25	56.74
S-13	542	0.29	0.04	0.24	37.95
S-14	568	0.30	0.04	0.25	32.91

^1^ Calculated with t-plot method. ^2^ Calculated with BJH method. ^3^ P/P^0^ =0.9905, 0.9908, 0.9918, 0.9923.

## Data Availability

The data presented in this study are available on request from the corresponding author.

## Supplementary Materials

The following are available online at https://www.mdpi.com/article/10.3390/nano11123171/s1, Scheme S1: (a) Material addition order through conventional method (b) Optimized material addition order, Figure S1: XRD spectra of samples S-6, S-9, S-10, and S-11, Figure S2: SEM diagrams of S-6 (a–c) and S-9 (d–f), Figure S3: SEM diagrams of S-10 (a–c) and S-11 (d–f), Figure S4: (a) N_2_ adsorption isotherm (b) pore size distribution of sample S-6, S-9, S-10, and S-11, Table S1: N_2_ physical adsorption data of S-6, S-9, S-10, S-11.
