# Supplementary material for: Growth Study of Hierarchical Pore SSZ-13 Molecular Sieves with Improved CO2 Adsorption Performance
Source: Nanomaterials (Basel). 2021 Nov 23;11(12):3171. doi: 10.3390/nano11123171 (PMC8704635; doi:10.3390/nano11123171)
Supplement: Supplementary file 1 [file nanomaterials-11-03171-s001.zip › nanomaterials-1471825 - SI for conversion- v1.pdf]

nanomaterials

Supplementary Material

Growth Study of Hierarchical Pore SSZ-13 Molecular Sieves with Improved CO_2 Adsorption Performance

Runlin Han [1,2,*], Yuxuan Tao [2] and Liang Zhou [2,3*]

[1] School of Chemistry and Chemical Engineering, Jinggangshan University, Ji'an 343009, China
[2] School of Chemical Engineering, Dalian University of Technology, Dalian 116024, China; tyxlogo@163.com
[3] Jiangsu Province Key laboratory of Fine Petrochemical Engineering, Changzhou University, Changzhou 213164, China
* Correspondence: author. hanrunlin@163.com (R.H.); zhouliang@dlut.edu.cn (L.Z.); Tel.: +86-4272631851 (R.H.)

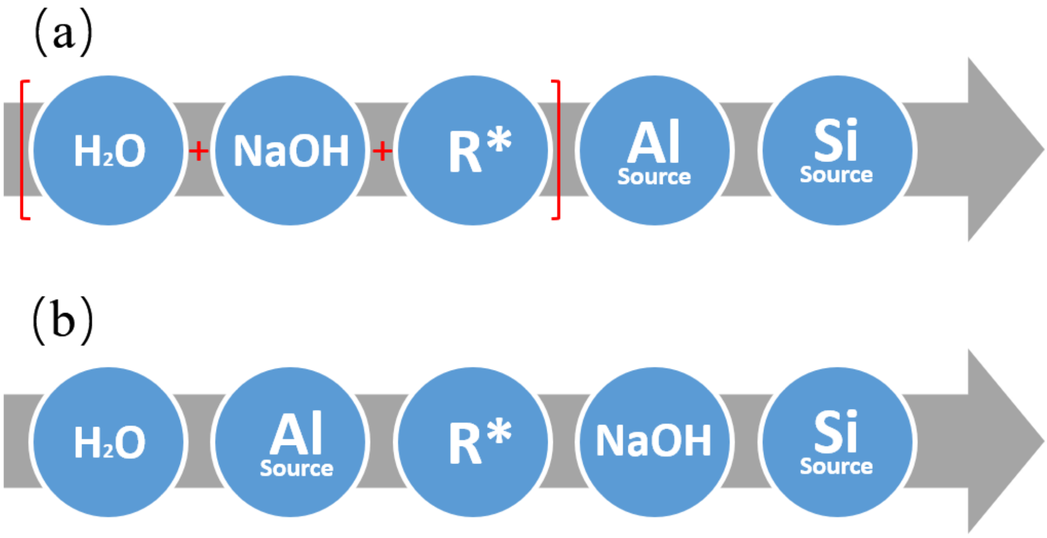

Scheme S1. (**a**) Material addition order through conventional method, (**b**) Optimized material addition order.

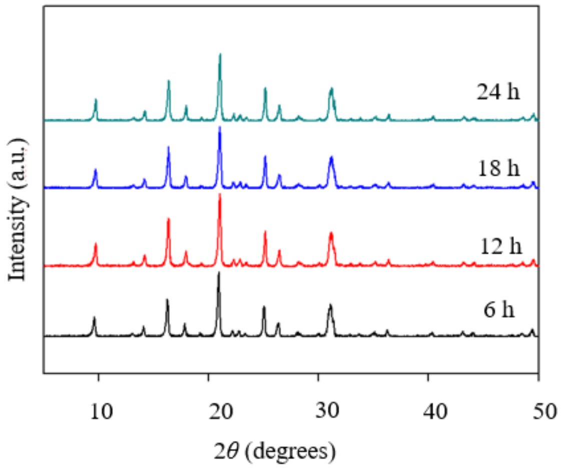

Figure S1. XRD spectra of samples S-6, S-9, S-10, and S-11.

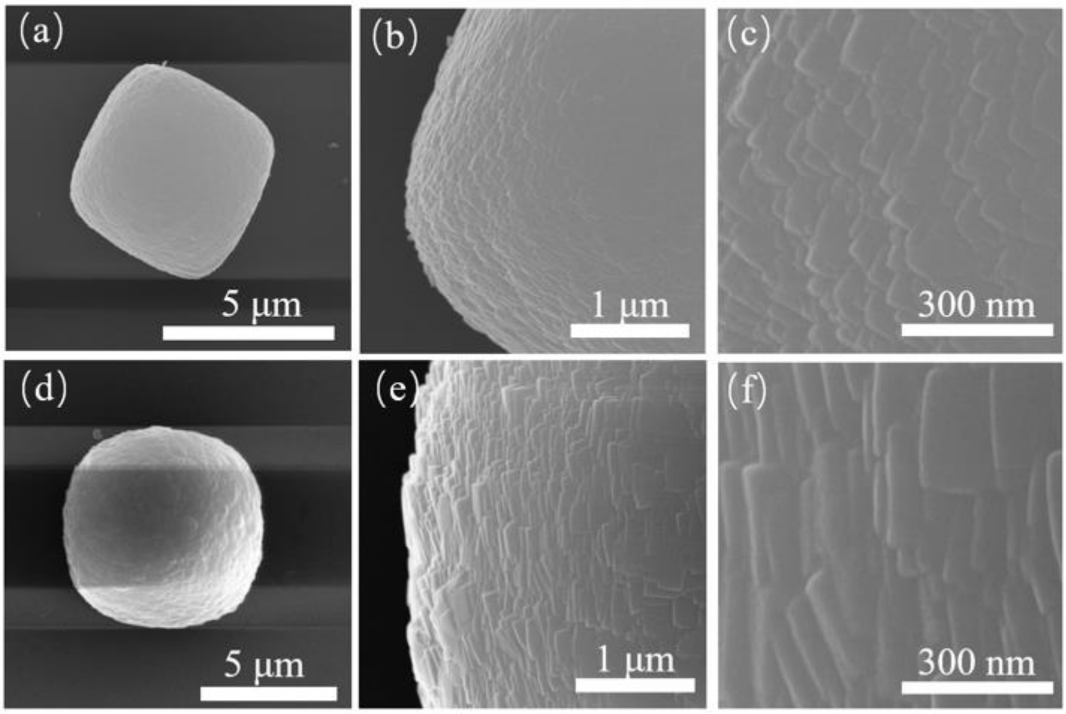

Figure S2. SEM diagrams of S-6 (**a**–**c**) and S-9 (**d**–**f**).

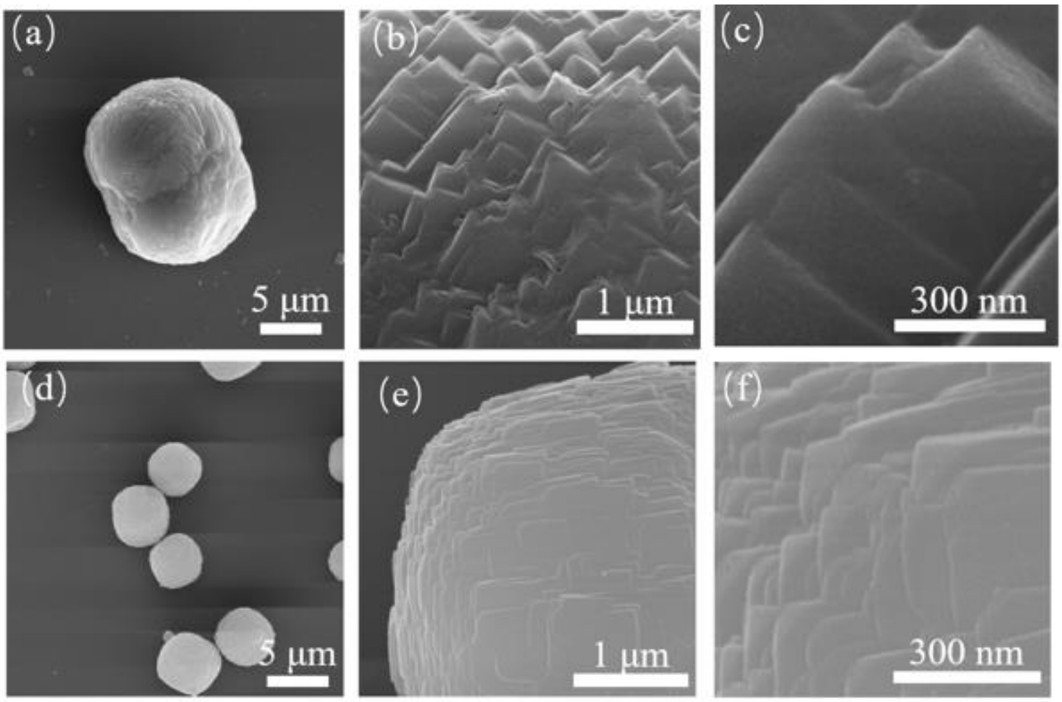

Figure S3. SEM diagrams of S-10 (**a**–**c**) and S-11 (**d**–**f**).

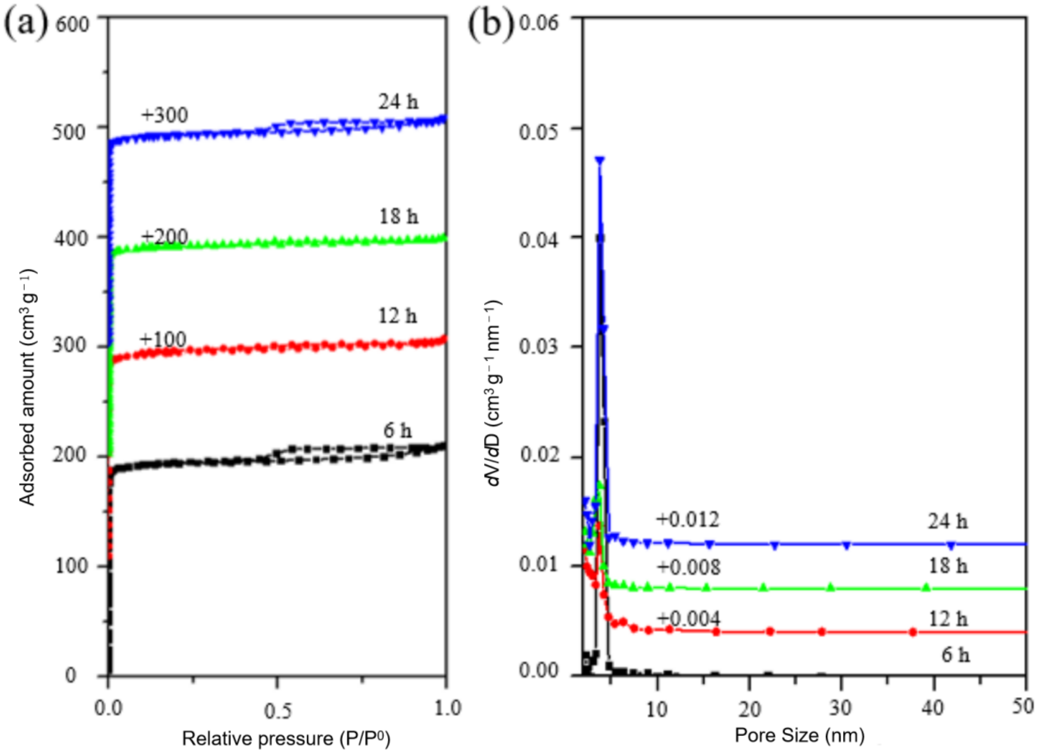

Figure S4. (**a**) N_2 adsorption isotherm (**b**) pore size distribution of sample S-6, S-9, S-10, and S-11.

Table S1. N_2 physical adsorption data of S-6, S-9, S-10, S-11.

Sample	S_{BET} (m^2/g)	Total Pore Volume [1,3] (ml/g)	Mesoporous Volume[2] (cm^3/g)	Micropore Volume [1] (cm^3/g)	Desorption Cumulative Surface area (m^2/g)
S-6	643.67	0.33	0.04	0.29	34.41
S-9	650.67	0.32	0.03	0.29	21.69
S-10	637.68	0.31	0.02	0.28	17.94
S-11	641.87	0.32	0.04	0.28	31.77

[1] Calculated with t-plot method

[2] Calculated with BJH method

[3] P/P^0 = 0.9904, 0.9909, 0.9895, 0.9905.
